# The Niebuhr net: A net for capturing benthopelagic fish and fauna with towed camera systems

**DOI:** 10.1016/j.ohx.2026.e00765

**Published:** 2026-04-03

**Authors:** Autun Purser, Axel Nordhausen, Ulrich Hoge, Tim Niebuhr, Loki Williams, Lisa Chakrabarti, Chiara Papetti, Malte Pallentin, Frank Wenzhoefer

**Affiliations:** a Alfred Wegener Institute, Helmholz Centre for Polar and Marine Research, Bremerhaven, Germany; b Max Planck Institute for Marine Microbiology, Celsiusstrasse 1 D-28359 Bremen Germany; c Richmond Upon Thames College, Twickenham, UK; d Russell Finex Ltd, Feltham, Middlesex, UK; e School of Veterinary Medicine and Science, University of Nottingham, Sutton Bonnington, Leicestershire, UK; f Biology Department, University of Padova, Padova, Italy

**Keywords:** Fishing net, Seafloor sampling, Benthic fauna, Benthopelagic fauna

## Abstract

Historically, assessing the flora of an area of seafloor has been made by physically sampling with a trawl net, with subsequent inspection of the collected animals. This approach to sampling does not quantify well the area of seafloor to be assessed, with nets deployed blind, and these trawls may cause extensive and long-term physical impacts on habitats. These impacts may be damaging, and therefore the approach inappropriate for use in assessing vulnerable areas, such as coral reefs or other niche ecosystems. Towed camera systems, capable of filming or photographing areas of seafloor facilitates the non-invasive estimation of the abundances of animals on or near the seafloor, but does not allow the collection of physical samples.

To address the drawbacks of physical net trawls and towed camera systems, the Niebuhr net has been developed. This simple device consists of a weighted net which can be mounted below a towed camera system, allowing an operator to monitor the lowering of the net to the seafloor directly, and to visually sample animals of interest. In this paper we present the design of the Niebuhr net as used by the Alfred Wegener Institute, Helmholz Centre for Polar and Marine Research, Germany, as well as results from an expedition with the research Icebreaker ‘*RV Polarstern*’ in 2025.

## Graphical abstract

**Figure:**
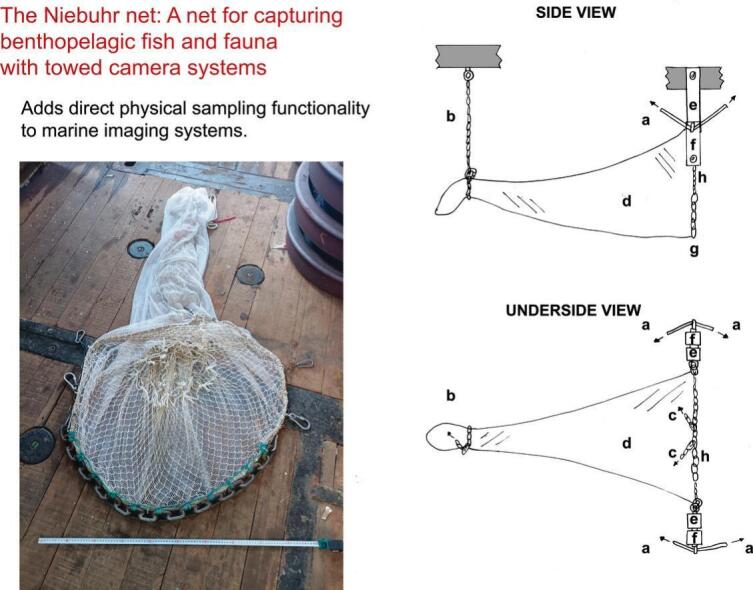

Specifications table.

**Table:** 

Hardware name	Niebuhr net
Subject area	• Biological sciences (marine biology / ecology) • Environmental, planetary and agricultural sciences
Hardware type	• Biological sample handling and preparation • Seafloor and lower water animal collection
Closest commercial analog	A more accurately targeted and lower seafloor impact alternative to the Agassiz trawl (https://www.kc-denmark.dk/products/dredges/agassiz-trawl,-300-x-80-cm.aspx)
Open source license	CC BY
Cost of hardware	400 euro

## 1 Hardware in context

Determining the distribution of fauna on the seafloor is important for many aspects of exploratory marine research, such as determining the benthic ecology of remote seafloor areas [1], [2]. Investigating the seafloor fauna inhabiting an area is also important for the determination of suitable sites for deployment of renewable energy systems such as offshore wind farms [3], for the oil and gas industry [4] or for gauging the impacts of pollution events [5], deep sea mining [6], other human pressures on the marine environment [7] or global climate change [8] on benthic communities. Traditionally, assessing seafloor fauna communities and distributions has been carried out via scientific trawling of the seafloor of interest [9], counting the numbers of animals collected within these trawls to compute abundances of animals over a known area of seafloor. These trawls collect animals living in the waters just above the seafloor (benthopelagic fauna [10]), those within the upper sediments (infauna [11]), and those living directly on the seafloor, either attached or crawling across it (epifauna [12]). An alternative methodology used has been to collect discreet samples of seafloor, usually up to 50 x 50 cm in size, with a ‘box corer’, ‘van veen’ or ‘multicorer’. These technologies collect fauna from a far smaller area of seafloor [13].

Scientific trawling, be it for investigating fauna in a new region or monitoring an area subject to pressure (natural or anthropogenic) will certainly impact on the seafloor topography, structure and ecosystem in general [14]. Keeping these trawls small, far smaller than commercial fishing trawls, can minimize the extent of these impacts, though the trawling approach remains unapplicable for areas of seafloor with a rough topography or particularly vulnerable seafloor communities, such as cold-water coral reefs [15]. Small ‘otter’ trawls and Agassiz trawls still cover considerable lengths of seafloor to catch the fauna required for analysis [16].

Since the 1950 s, various attempts to collect underwater TV, video or still images of the seafloor, to both quantify fauna and investigate ecosystem interactions without direct disturbing interference hve been ongoing [17], with the current generation of towed video / still image camera systems capable of collecting HD video and still images using fibreoptic cable winched from a ship for many hours at a time, in some cases whilst simultaneously collecting sidescan bathymetric seafloor topographic data [18]. These systems are particularly useful for monitoring environmental / fauna change over time [8], [19], [20], and will likely show progressive changes of seafloor community structure as regions of the ocean continue to warm, or in response to potential impacts of new anthropogenic stressors, such as deep sea mining [21], [22]. What they cannot do is collect physical voucher specimens from areas explored for the first time, or of animals migrating into a region over time, which can then be used to compare with museum collections or for consultation with taxonomists to identify species unambiguously [23], [24]. Recent developments in identifying the use of areas of ecosystems from environmental DNA (eDNA) is further boosting the requirement for limited collection of fauna directly from the environment for eDNA results validation [13].

Seafloor trawling, with nets fitted with video cameras viewable by operators on a surface vessel can reduce somewhat the blindness of trawl sampling (video trawling), though large areas of seafloor are still significantly disturbed by such systems [25]. Smaller samples can also be collected with a ‘TV or video grab’, basically a box corer with a live feed which can be used to collect a discreet area of seafloor, usually less than 1 m^2^ [26]. An advantage a video trawl has over a TV grab is the facility to collect mobile fauna, such as fish, which are extremely difficult to target with vertically deployed ‘TV or video grab’.

The Niebuhr net, here presented, was recently developed by the Alfred Wegener Institute, Helmholz Centre for Polar and Marine Research (AWI) to combine the strengths of video trawl sampling (large area surveyed and ability to collect mobile animals such as fish) with those of video sleds (direct imaging of seafloor) whilst minimizing the negative aspects of both. The net was developed to be used in conjunction with the research icebreaker *RV Polarstern* [27] and the Ocean Floor Observation and Bathymetry System (OFOBS) towed camera sled [18]. The Niebuhr net allows an operator to lower to or raise the net from the seafloor rapidly, allowing direct collection of fauna samples with a far reduced impact than can be expected from TV trawls, whilst also allowing still image and video collection (video sled data) when held in both the ‘active collection’ (in contact with seafloor) and ‘passive observation’ (filming seafloor from ca. 1.5 m height) modes. This net supersedes the small ‘Agassiz’ benthic trawl nets [28] favored at present for seafloor benthic fauna collection in ‘at risk’ or discreet seafloor areas where fauna monitoring is important, and where sampling impacts are to be minimized. Constructed with a range of off the shelf components the net can be fabricated and modified cost effectively to fit any towed camera system, utilizing a novel arrangement of passive elastic control cords to maintain stability even under fairly complex vessel motion and seafloor structure conditions.

## 2 Hardware description

### 2.1 General description

This work describes the design and construction of a Niebuhr net; a benthic sampling net to be mounted directly onto a towed camera sled, and which is capable of collecting seafloor and epibenthic / lower water column fauna from any depth. The net offers several advantages over other direct seafloor fauna sampling methodologies, primarily it allows direct inspection of the seafloor target area by the operator, and simultaneous video or still image capture of the seafloor and target fauna.

**Fig. 1:**
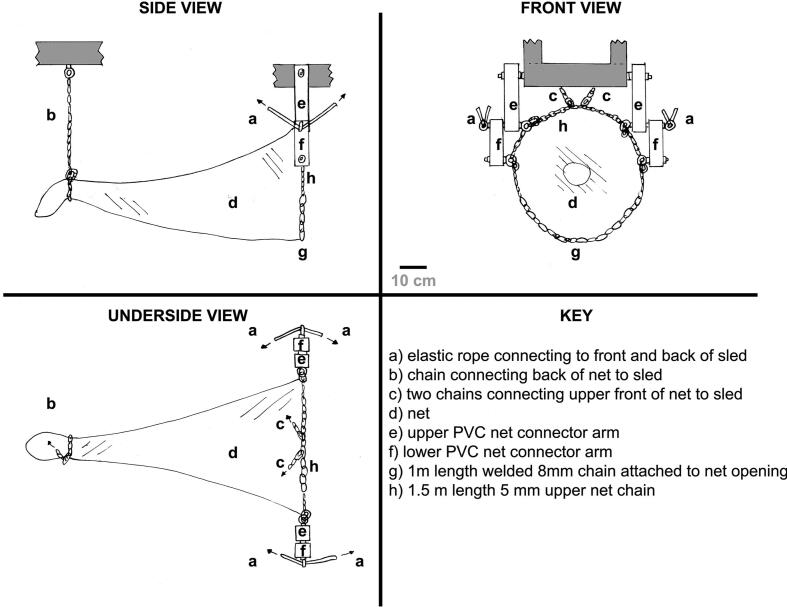
Schematic of the Niebuhr net.

**Fig. 2:**
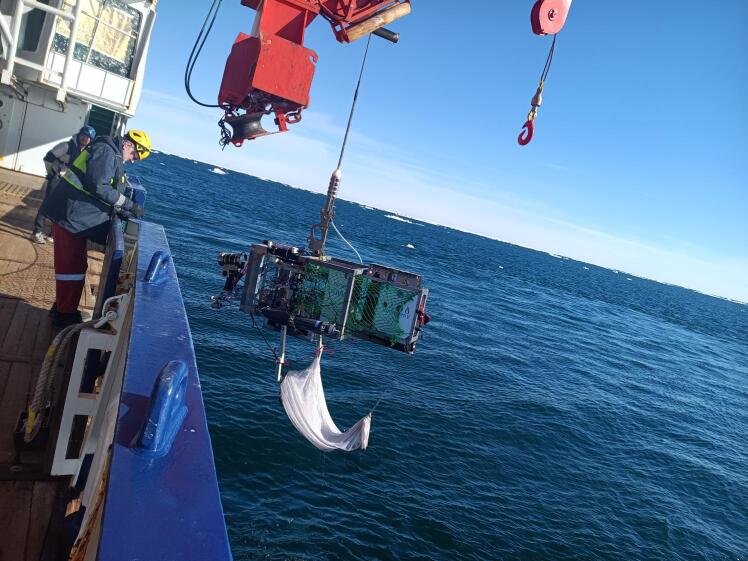
Niebuhr net attached to the AWI OFOBS towed camera system during the PS146 expedition to Antarctica, Feb 2025.

**Fig. 3:**
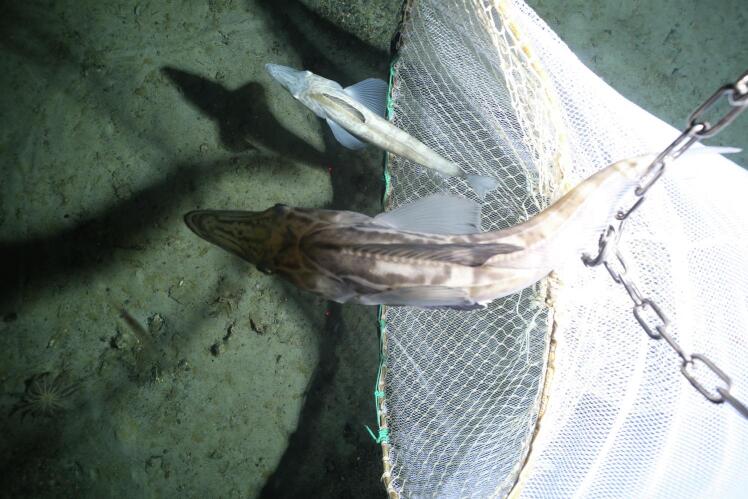
The Niebuhr net at collection depth on Jan 29th 2025 within the Weddell Sea, Antarctica at a depth of 550 m. Two *Neopagetopsis ionah* benthopelagic icefish can be seen swimming ahead of the net. The lower fish was caught by the ‘Niebuhr net’ as it was towed, the upper icefish was above the net opening and therefore evaded capture. The image was captured directly with the AWI OFOBS camera sled, to which the net was affixed for the PS146 expedition.

The net consists of a dual layer net, with a coarse inner net for separation on collection of larger fauna (typically fish) and a finer outer net for collection of smaller epifauna, such as sea urchins, starfish etc. Both these nets are connected to a chain weight at the collection end, two heavy plastic articulated arms connected to the video sled and two chains also connected to the sled to keep the upper opening of the net open, and to keep the tail, collection end of the net from dragging too much on the seafloor or getting tangled up with the video sled on deployment. Another crucial component of the Niebuhr net is the presence of elastic cords running between each mounting arm and the front and back of the video sled, with these cords passively ensuring that the net remains vertically below the video sled, and that the opening of the net retains its position under the camera of the video sled during use, regardless of the complexity of the seafloor. The schematic of the net is given in Fig. 1, with it mounted and attached to a towed camera sled, in this case the AWI OFOBS system [18]. The entrance to the net, and the two parallel plastic arms, are placed level with the towed camera sled camera port. This allows the sled operators to see both what is in front of the net on and above the seafloor, and whether or not a target of interest has passed into the net. Fig. 2 shows the net on recovery to deck (*RV Polarstern*, in the Weddell Sea, Antarctica in January 2025), with Fig. 3 showing the net during a collection deployment (see section 7 for validation information).

The Niebuhr net can be mounted directly below most towed camera sleds, adding the additional functionality to a deployment:

• Net expands the functionality of a towed camera sled, to also allow the video or imaged collection of physical fauna specimens directly from their home ecosystem.
• The small size of the net results in a far lower seafloor disturbance footprint associated with benthic fauna collection than may be expected with epibenthic sled use or scientific trawling.
• The net requires a weighted chain link rather than the heavy metal frontispieces associated with the majority of benthic sampling equipment. This reduces both construction and shipping costs of the device.
• By mounting the Niebuhr net directly on a towed video sled expedition time can be saved by it facilitating both direct physical sampling and habitat imaging during the same deployment.

The Niebuhr net consists of two main sets of components; the net itself, and the attachment mechanism to secure the net to the camera sled with which it will be used. These are discussed below.

### 2.2 The net

**Fig. 4:**
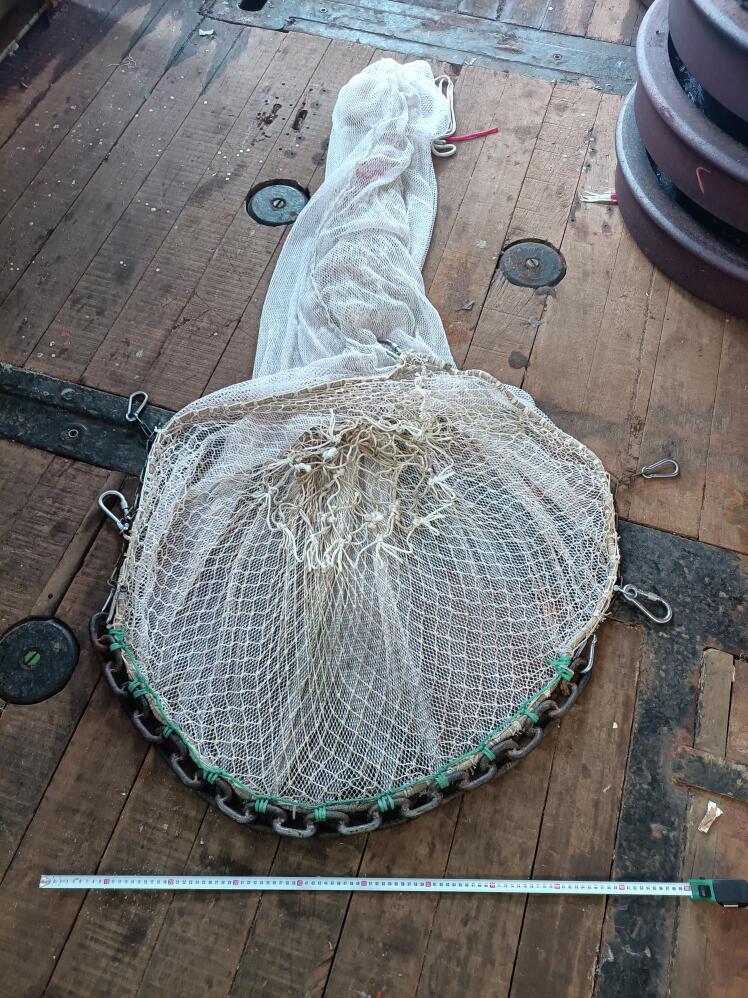
Photograph of the Niebuhr net on the deck of the research vessel *RV Polarstern* during the PS146 expedition.

The Niebuhr net was designed to collect benthopelagic and benthic megafauna by being towed below a video sled, itself towed by a research vessel. The net was designed specifically to collect adult *Neopagetopsis ionah* icefish (ca. 50–60 cm length), fish which guard egg colonies in Antarctic waters as part of their lifecycle, and also to collect the eggs (ca. 1 cm diameter per egg) [29]. To be able to collect two targets with such disparate sizes required the use of a double layered net (Fig. 4). The outer layer of the net is a narrow hemp weaved net with a 0.5 cm weave size. The inner net is a hemp net with a 4 cm weave size. This double layered net comprises two nets of conical shape, with the wide aperture, the front of the net, having a diameter of 1 m, a length of 2 m, with the taper with length down to ca. 20 cm diameter. The front aperture comprises a 10 mm wide nylon rope attached by a circular cord ring connected to the apertures of both nets, and a weighted, welded 1 m long, 8 mm diameter metal semicircle chain to give the lower form of the net. The semicircle form was selected as it was a useful shape to allow the net to drop into *Neopagetopsis ionah* nests – which are depressions of similar dimensions. Variations of both the size and shape of the weighted section of the net can be made to facilitate use in different environments, though the shape also proved very useful for collecting fauna from the seafloor in both flat and rocky areas of the seafloor, as well as the icefish nesting areas. The upper semicircle of net is also tied to the nylon rope, whilst also being tied to an unwelded, lighter chain matching the remaining circumference of the entrance circle (5 mm thick chain links). Within the central aperture, a ‘non-return’ ring of material is stitched into the net entrance, to prevent egress by fauna which have passed successfully into the net. This non-return ring is made from offcuts of the 4 cm inner net hemp material Fig. 4). The back of the net is an open, narrow 20 cm, with both nets knotted together and secured with a rope during deployments, which can be opened on retrieval of the system to the research vessel after target capture, to allow collection of the fish / other fauna. (The layout of the net is given in Fig. 1).

### 2.3 The attachment mechanism

Towing a net across a potentially rocky seafloor comes with the risk of the net getting caught, or the net always pulled back and the open aperture flat to the seafloor as the device is towed forward. Several features of the Niebuhr net mitigate against these eventualities. Firstly, the net is attach at its open end to two joined arms hanging vertically from the camera rig, and attached to it by a pair of metal bars perpendicular to the direction of towing. These arms are jointed in the middle, with carabiners attaching at two points on either of these arm pairs, as shown in Fig. 1. These arms are loose and free to move forward and backward, both at the point of connection with the towing device, but also at the joining point between the two arm parts on each side of the net. This allows the net some mobility to swing back and forth, and independence on either side of the tow line. The weight of the lower welded chain section on the open aperture at the front of the net keeps the net open when the device is hanging. To stop this section dragging when the device is in contact with the seafloor, a pair of nylon bungee ropes attach from the bottom of each arm pair to both the front and back of the towing sled. These nylon ropes are tied taught. These bungee ropes are of appropriate length to ensure that the forces at work (the tension to the front and back attachment positions = keep the net straight and vertical below the towing sled, whilst allowing the appropriate ropes to stretch if a rock or boulder happens to touch the net – allowing the open net aperture to swing with the contact. After the rock has been passed, the tension in the bungee ropes allows the arms to revert to the initial vertical position. These bungee attachments are shown in the Fig. 1 schematic and can be seen in action on the OFOBS towed sled in Fig. 2. These bungee ropes maintain a certain rigidity / flexibility. Two chains connect the upper loose chain of the net open aperture chain to the towing device with a pair of carabiners. A further chain is attached to the back of the net, attached by carabiner to the sealing rope used to keep the narrow end of the net closed during deployment, but readily accessible on retrieval to the research vessel (Fig. 1).

## 3 Design files summary

**Table:** 

**Design file name**	**File type**	**Open source license**	**Location of the file**
Net design	.jpeg	GPL	Available with the article Fig. 1_HardwareX.jpeg

### 3.1 Net design figure description

**Net design** – A technical drawing of the Niebuhr net (Fig. 1), as mounted on the OFOBS video sled [18].

## 4 Bill of materials summary

**Table:** 

**Designator**	**Component**	**Number**	**Cost per unit −currency**	**Total cost −** **currency**	**Source of materials**	**Material type**
N1	*Net outer (*ca. *0.5 cm weave)*	1	54.95 euro	54.95 euro	https://tinyurl.com/4hceym7m	Hemp
N2	*Net inner (*ca. *4 cm weave)*	2	16.45 euro	32.90 euro	https://tinyurl.com/ywunvyrz	Cotton
C1	*10 m 100 mm diameter rubber bungee rope*	1	16.77 euro	16.77 euro	https://tinyurl.com/mvaujdb8	Rubber
C2	5 m 5 mm stainless steel chain to attach net to camera sled	2	21.59 euro	43.18 euro	https://tinyurl.com/ymv6bc29	Metal
N3	2 m 8 mm Stainless steel bottom weight chain	1	51.41 euro	51.41 euro	https://tinyurl.com/5pfzk4jv	Metal
C3	M12 ring nut A2 steel	12	5.24 euro	51.48 euro	https://miniurl.com/xao2j17c	Metal
C4	M12 1 m threaded steel rod	1	2.50 euro	2.50 euro	https://miniurl.com/EmetalRing	Metal
C5	DIN 985 M12 Locking nut	25	0.41 euro	10.25 euro	https://tinyurl.com/3jptkmwa	Metal
C6	M12 Washer DIN-125A	25	0.20 euro	5.00 euro	https://tinyurl.com/nbpxa6us	Metal
C7	PVC 6 cm thick sheet, 12 cm x 100 cm	1	253.79 euro	253.79 euro	https://tinyurl.com/yvjfnvm6	PVC plastic
N4	30 m nylon paracord (3 mm)	1	14.99 euro	14.99 euro	https://tinyurl.com/4j56h7vn	Nylon
N5	10 m nylon rope (10 mm)	1	12.35 euro	12.35 euro	https://tinyurl.com/yrp86j96	Nylon
C8	M8 steel screw lock carabiner (2 pack)	5	7.99 euro	39.95 euro	https://tinyurl.com/7yy47t3w	Metal
N6	Stainless Steel Screw Shackles (M8 6 pack)	1	10.99 euro	10.99 euro	https://tinyurl.com/ycyt4pak	Metal
N7	Pack of 100 450 mm x 9 mm cable ties	1	16.99 euro	16.99 euro	https://tinyurl.com/3u2bpw25	Plastic
S1	140 g/m^2^ – 3 x 3 m tarpaulin	1	15.99 euro	15.99 euro	https://tinyurl.com/3bywpsuu	Plastic

## 5 Build instructions

### 5.1 Net construction

The Niebuhr net has been designed to hang below a camera sled in close proximity to the seafloor. The OFOBS camera sled [18] is deployed 1.5 to 2 m above the seafloor, so a net of 1 m diameter was considered optimal. The build instructions here are therefore for building a net with a front opening diameter of 1 m and a length of 2 m.

**Fig. 5:**
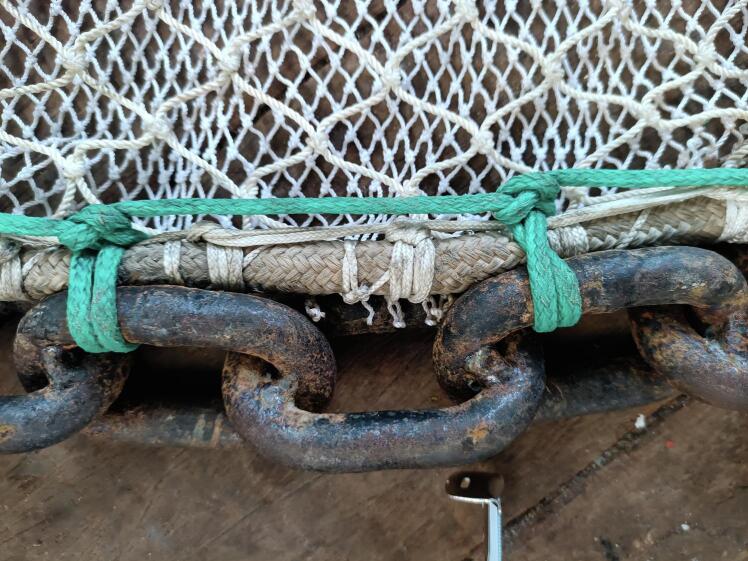
Close up of a section of the Niebuhr net where the two net layers are lashed to the nylon circular opening circumference rope and a portion of the tig-welded heavy lower chain.
i) After lashing the nylon rope to the two net openings, to permanently join the front open ends of the nets to each other, lash with heavy duty nylon the (cooled) welded, curved bottom weight chain produced in stage g) (Fig. 5).
j) Four carabiners (C8) should now be attached to sides of the entrance chain of the Niebuhr net to allow it to be attached to the lower, free-swinging section of the PVC attachment arms. These should be spaced to two pairs on each side of the net, approximately 30 cm apart from each other and about 30 cm from the ends of the weighted chain lower section (see Fig. 4 for arrangement).
k) Use an appropriate length of the thinner chain (C2) to match the remainder of the entrance aperture rope, cutting to length with bolt cutters. This will be approximately 1.9 m in length. Use two shackles to attach the ends of the thinner chain to the ends of the thicker chain.
l) Use the thinner nylon cord to lash each link of the thinner chain to the two nets and nylon rope loop in the same manner as used in i).
m) Cut a circle of ‘Net inner’ material of 0.6 m diameter. Within this circle cut out and remove a circle of 0.3 m diameter.
n) Lash the exterior of the circle of material approximately 60 cm into the inner net layer to the inner net layer. This forms the non-return component of the net, to prevent fish which have swum into the net or been caught from easily escaping.
o) Use 6 cable ties (N7) roughly 0.8 m into the nets to connect the nets loosely at the mid-point. By lightly closing the 450 mm cable ties between two net mesh segments (one from each net layer) every ca. 60° around the net pair, these cable ties will prevent the two layers collapsing on each other fully during deployment, and will also aid in increasing the functionality of the non-return component.
p) Tie 1 m of nylon rope securely approximately 20 cm from the narrow end of the net layers. Good practice is to weave in the folded over remaining 20 cm of nets into one of the rings of this securing rope. This rope secures the collected fauna during deployments but can be opened to release the collected fauna at the end of a deployment.

a) Cut an appropriately sized triangular piece of the ‘Net Outer’ material (N1). Given a net diameter of 1 m and length of 2 m, this will be an isosceles triangle with a base of 3.14 m and a narrow end tip of 20 cm diameter.
b) Carefully weave the two long edges of the outer net triangle together using thin nylon paracord (N4). This is best done with an overlap of 6 of the small net spacings all the way along the net. A double weave line of two parallel paracord lines running the length of the net will strengthen the net.
c) Repeat a) and b) with the ‘Net inner’ material (N2). Maintain an overlap of 4 of the 4 cm net spacings. This will result in a net layer slightly narrower than that produced in steps a) and b).
d) Insert the inner, wider spaced net into the outer net so that the two openings are lined up. Do this in a large open area of floor or on a large table so the two net layers can be laid flat, one inside the other.
e) Use approx. 3.5 m of nylon rope (N5) to form and tie a loop of the circumference of the large opening of the two nets (3.14 m circumference, with the remaining length used to knot the loop.
f) Lie the loop on a surface suitable for tig-welding and form it into a circle.
g) Place 1.2 m of the 8 mm stainless steel bottom weight chain (N3) alongside the rope circle loop. Remove the loop from the working area and tig-weld (under standard workshop safety protocols; safety goggles, clothing etc. Under the supervision of lab manager or welding professional) each link to render the curve permanent.
h) Lash the two net openings to the nylon rope, placing one lashing through each of the ‘Net inner’ net mesh spacings, and every appropriately spaced mesh spacing from the outside net (see Fig. 5).

### 5.2 Attachment mechanism construction

Not all camera sleds are directly comparable. The OFOBS system used by AWI has a diameter of 95 cm, and therefore the dimensions of the Niebuhr net and attachment points were derived from this measurement. Slightly wider or narrower camera sleds will require some modification in the Niebuhr net and attachment mechanism design, but a key point is that the arms of the attachment mechanism, connecting the net (section 5.1) to the camera sled should be placed so that the net opening hangs halfway across the images which will be collected by the camera, as in the example image given in Fig. 3. These instructions are to construct an attachment mechanism suitable for mounting a Niebuhr net onto on camera sled of ca. 2 m length, 95 cm width.

a) Cut the PVC sheeting (C7) into 4 PVC bars. Two of these bars should measure 60 x 6 x 6 cm, with two cut to measure 40 x 6 x 6 cm. Cutting should be carried out by skilled technicians following workshop safety protocols.
b) 3 cm from the end of both ends of both bars, holes should be drilled to allow free movement of a M12 rod (C4) through their centre.
c) Six lengths should be cut from the C4 metal rod. Two of these need to be of sufficient length to pass through the PVC bars, three washers (C6), a locking nut (C5) and have sufficient additional length to be attached to the camera sled. How this attachment will be achieved will depend on the sled. For the validation deployments discussed in section 7, this was done by passing the C4 rods through PVC blocks mounted permanently on the camera sled, and with these bolted firmly to this.

The second two pieces of C4 rod should be of sufficient length to pass through both rods, three washers (C6), two ring nuts (C3). The third pair of rod lengths should be of sufficient length to pass through one PVC length, two washers (C6), one locking nut (C5) and a ring nut (C3).

d) The longest pair of PVC lengths should now be bolted onto the towing sled. This should be done by attaching the appropriately cut C4 rods to the sled, then placing a washer (C6) onto the bar, followed by one end of the PVC rod, another washer (C6) and then a locking nut (C5). The bar should be able to swing with one direction of freedom (back and forth in and away from direction of tow travel). Both long PVC lengths should now be attached, one on each side of the towing sled.

How the sled and attachment should be carried out, whether on a crane or flat on a workroom floor, will depend on the design of the towing sled. For the OFOBS sled discussed in validation section 7 the sled was placed on a workshop floor for all the following steps. If a crane is required, care and experienced technicians should direct the work.

e) The shorter PVC lengths should now be attached on the ends of the longer PVC lengths not already attached to the towing sled. This should be done by placing the shorter rods on the outside and parallel to the longer PVC lengths, so the drilled holes line up. Then, the appropriate lengths of C4 rod should be placed through the holes. This should be done by placing a M12 ring nut on the inside (C3) followed by a washer (C6), the first, shorter PVC length, a second washer (C6), the second, longer PVC length, another washer (C6) and finally another M12 ring nut (C6). This assemblage should allow the second PVC bar to pivot at the point of connection with the upper PVC bar.
f) Three lengths of 5 mm chain (C2) should now be cut with bolt cutters. One of these should be of sufficient length to allow the Niebuhr net back, narrow point to be attached at the closure rope to the rear of the towing sled. One end of the chain should be attached with a carabiner(C8) to the knotted rope closing the nets. The other end should be attached to the towing sled via a method which will be different depending on the towing sled design. For the validation described in section 7, a ring nut was affixed to the towing sled at its rear and a carabiner (C8) used to attach this to the chain. The chain should be of a length to position the rear of the net a little below the midpoint of the net opening during towing.
g) Two shorter lengths of 5 mm chain (C2) should be cut to allow the upper semicircle of the Niebuhr net to be attached to the towing sled. These two lengths should be sufficient to hold the upper portion of the net entrance open, so it doesn’t collapse down over the weighted lower section during deployments. The position and mounting arrangement of these nets will vary with towing sled, but for the validation described in section 7 these two chain lengths were carabinered to ring nuts affixed to the towing sled and to two points on the net entrance chain (see Fig. 4).
h) 4 lengths of elasticated bungee rope should now be tied to the exterior ring nut marking the junction between the upper and lower pieces of the PVC arms on either side of the camera sled. These lengths should be sufficiently long to allow the end points to be attached to the front and back of the towed sled (at points which may vary by towed camera device), and sufficiently tightened to keep the net entrance vertical during deployments. See section 6 for the attachment of the bungee ropes to the camera sled.

## 6 Operation instructions

### 6.1 Attaching net to towing sled

Towed camera sleds are generally winched from the deck of research vessel some few meters, then lowered into the ocean via an A-frame or crane on the deck. When a Niebuhr net is to be fitted to a towed sled for such a deployment, the following steps should be followed:

a) The towed sled should be connected to the ships winch cable and video feed connectivity systems. This will vary according to device. The regular pre-dive testing of the camera systems should be carried out before attaching the Niebuhr net.
b) The towed sled should be winched a small distance above the ships deck to allow attachment of net.
c) The four carabiners mounted on the sides of the net should be attached to the four interior mounted ring nuts on the PVC arms of the mounting apparatus already attached to the camera sled.
d) The two chains carabineer to the upper chain of the net entrance should be attached to the towed camera frame the length adjusted if required to allow the net to hang vertically with aperture open below the towed camera system.
e) The rear chain attached to the closed collection end of the net should be attached to the rear of the camera sled, and adjusted in length to ensure the rear hangs slightly lower than the front opening of the net.
f) If not already done so, the towed camera sled should be winched to a sufficient height to allow the net opening to hang vertically down so it is visible in the towed camera live camera feed by the operator.
g) The four bungee ropes attached to the exterior ring nuts (2 on each side of the towed camera sled attachment arms) should now be connected to the front and back of the camera sled. The tension in these bungee ropes should be manually adjusted by varying the know used until the PVC arms of the attachment apparatus are hanging vertical, but held under tension. If the arms are pushed or pulled, they should allow the net to swing one way or the other, but after the pushing or pulling force is removed these arms should swing back into the initial vertical position.

The Niebuhr net is now correctly attached to the camera sled and ready for deployment.

### 6.2 Deployment of towing sled and net

After attaching the net to the camera sled, deployment and winching to depth can be carried out as follows.

a) The towed sled and underslung Niebuhr net should be winched off the ship and into the waters to be surveyed following standard operational procedures for the deploying research vessel. Additional care should be taken if there is any ocean movement as waves or swell may interact with the net. Visually the net should be inspected from deck until it is lowered fully into the water and lowered at a speed of max. 0.2 m s^−1^ to a depth of 5 m.
b) When the net and tow camera are under water the pair can be lowered to ca. 5 m above seafloor following standard operating parameters for towed camera platforms on the particular deploying research vessel. If possible the towed camera should be started during descent and the opening of the net manually inspected to make sure it remains below the towed camera during deployment and does not get overtaken by the lowering platform and entangled with it.
c) The towed camera and net should be lowered at a speed of 0.3 – 0.5 m s^−1^ to 5 m above seafloor. If visual inspection is possible and the net appears to be swinging upward and getting potentially overtaken by the lowering camera sled, then lowering speed should be reduced.
d) On reaching an altitude above the seafloor of approximately 5 m (as determined by towed camera platform specific methodologies, be these simple video inspection, inspection of lazer point spacings, Ultra Short Base Line (USBL) transponders or a combination of all of these [29], then the sampling procedure can be started.

### 6.3 Conducting tow sampling

After the camera sled and net has been lowered to an altitude of ca. 5 m above seafloor, the net will be hanging to a height of ca. 3 m above the seafloor. At this point the tow can commence.

a) A heading for the direction of tow is given to the bridge of the research vessel or pilot of the vessel for smaller ships. An appropriate speed is selected – ideally 0.05 – 0.3 kts.
b) The seafloor is inspected by the camera operator as the ship begins to move. Half of the camera feed is taken up with the net, which should be hanging near vertically below the camera – perhaps slightly pulled back away from direction of travel as movement starts.
c) If the camera operator sees fauna of interest, he/she may lower the net, so that the net opening is positioned to likely catch the fauna of interest with the weighted, welded bottom chain of the net allowed to drag across the seafloor. This will ideally allow the fauna of interest to be scooped into the net successfully.
d) Throughout a tow, video or image data can be recorded throughout, on timer or be operator driven to record captures – whichever approach is most applicable to the research program.
e) Items and fauna passing over the bottom weighted net chain should pass through the non-return ring and remain within the nets. Fauna larger than 4 cm will be retained within the inner net whereas smaller fauna will fall through and be retained by the outer net.
f) After the attempt has been made to catch an animal or item of interest the net can either be raised by ships winch some cm into the water column, or remain in contact with the seafloor if a general benthic sample is required. If the seafloor is particularly soft and the speed of tow slow, or benthic flow conditions of a particular nature or velocity, resuspended particles may be elevated into the water column by ground contact with the weighted net chain. If this is the case the net can be hoisted some cm into the water column and maintained out of contact with the seafloor until the net is towed out of the resuspended material cloud.
g) If large boulders or rough, rocky terrain is observed approaching, the net can be hoisted to an appropriate altitude above the seafloor to avoid possible entanglement or snagging. The PVC attachment arms however, with the bungee rope elasticity, allow the net to come into contact and slide over the majority of encountered obstacles without concern. If it is of research interest, pebbles and small rocks can also be directly collected with the Niebuhr net, as well as any attached epifauna.
h) The net is of sufficient size for numerous attempts to collect fauna to be made. A duration of several hours has been tested, with a varied and diverse collection of organisms made. The slow speed of Niebuhr net deployment, the limited ground contact and the separation out of the organisms by size leads to a sampling of fauna in good condition, often remaining alive until reaching the research vessel (depending on how steno bathyal a target animal is).
i) After the operators have decided a sufficient duration of time or number of fauna have been acquired, the tow sampling effort can be concluded and the tow camera and net hauled back to the research vessel.

### 6.4 Return to deck and sample collection

After a sufficient sampling effort has been made and the operators are satisfied with the deployment and are ready the tow, the following stages are ideally followed:

a) The bridge or ships pilot are informed that the tow is completed and that the equipment will be winched to deck.
b) The ship reduces speed as the camera platform and Niebuhr net are winched back toward the deck. Since the net is below the camera platform the system can be winched out of the water at a faster rate than it was lowered, at a speed of perhaps 0.5 – 0.7 m s^−1.^
c) For some collected fauna, pressure change may be a significant problem if living specimens are required. In these cases, if a collected animal is thought to be steno bathyal across a narrow range of pressures, it might be beneficial to pause the hoisting of the sled from time to time to allow the collected animal(s) time to adjust to the decreasing pressure. This will depend on the target fauna and research questions being investigated.
d) On breaking the surface, the camera sled should be hoisted high above the working deck if possible, to allow access to the underslung net.
e) The 3 m x 3 m tarpaulin (S1) should be placed underneath the Niebuhr net and camera platform on the deck.
f) The Niebuhr net should be detached from the camera platform. This should be done by disconnecting the following carabiners – in order – the two chains connecting the upper net opening chain to the sled, the four carabiners connecting the PVC arms to the net and finally the rear chain connecting the narrow end of the net to the rear of the camera sled. As these carabiners are undone, the net should be lowered into the center of the tarpaulin.
g) The tarpaulin should be folded over the net and then pulled out of the way of the camera platform.
h) The camera platform can then be lowered to deck and the PVC arms held up and out of the way with pieces of bungee rope attached to the attachment arm ring nuts, perhaps with the rope pulled taught across the top of the camera platform.
i) After the camera platform is stowed on deck, the catch can be investigated.
j) The rope holding the narrow end of the net shut can be removed. This should allow access to any larger fauna caught during a deployment, for example fish or squid. These can be placed into buckets of seawater directly if they are intended to be kept alive, or preserved as required. Any other fauna collected within the two net layers can now be separated and preserved / stored as required. Any dropped or escaping fauna will likely remain on the tarpaulin and be easy to collect again.
k) Once the rear end of the net is cleared, the rest of the net can be inspected. Spiky fauna, such as starfish or echinoids, or some fish, can be entangled closer to the net entrance opening and may require careful manipulation to extract for storage etc.
l) Once all the collected fauna or items have been collected, it is good practice to wash everything down with freshwater, to aid in maintaining an extended usability longevity.

## 7 Validation and characterization

### 7.1 The RV Polarstern PS146 expedition in the Weddell Sea (Antarctica)

From December 2024 to March 2025 the research icebreaker *RV Polarstern* carried out multidisciplinary research work in the ice covered Weddell Sea region of the Southern Ocean, Antarctica [30], the PS146 expedition [31]. A component of this work was to continue the visual characterization of the Weddell Sea seafloor, an ongoing research topic for the Alfred Wegener Institute [32], [33] and to collect eggs and fish from the vast nesting *Neopagetopsis ionah* icefish colonies discovered during the PS124 expedition in 2021 [29], using the Niebuhr net – the task for which the net was invented. Unfortunately, the timing of the PS146 expedition did not correlate with active icefish nesting. Even so, during the expedition the net was used to collect fauna from 11 locations in the Weddell Sea.

### 7.2 The use of the Niebuhr net in the Weddell sea

The Weddell Sea is a large component of the Southern Ocean in Antarctica, and is primarily ice covered throughout each year [34]. The seafloor of the Weddell Sea is of depths of 10 s of meters to several thousands of meters [30], but is all within the range of use of the AWI OFOBS towed camera system [18] (6000 m) to which the Niebuhr net was attached for its trial deployments. To operate in this densely ice packed environment, during the PS146, the *RV Polarstern* smashed the ice for deployments and towed the camera sled with the Niebuhr net attached, behind the vessel at a speed of 0.2 – 0.5 kts.

**Fig. 6:**
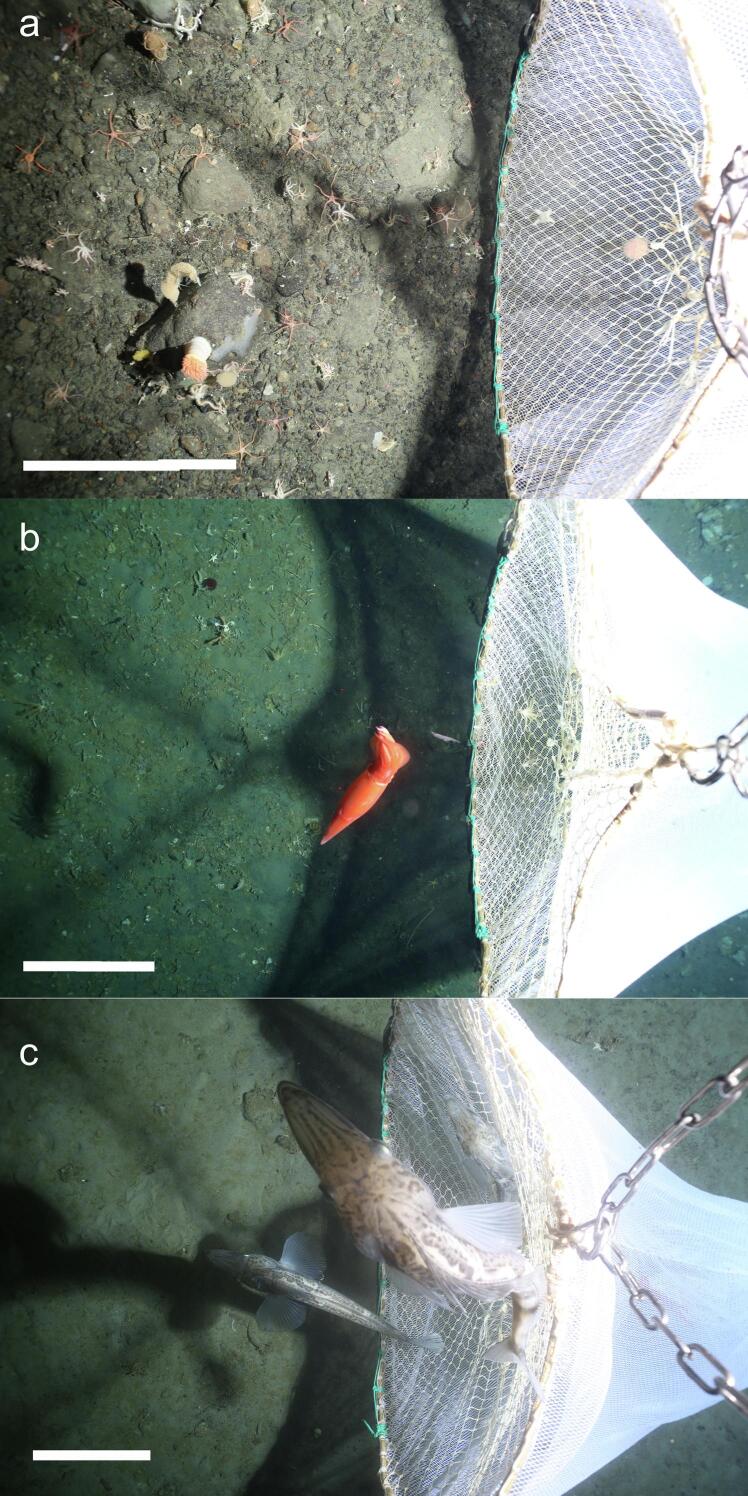
Three images of the Niebuhr net being used for physical sampling (in conjunction with the AWI OFOBS camera sled) during the PS146 *RV Polarstern* expedition to the Southern Weddell Sea, Antarctica, Jan – Mar 2025. In all cases the sled is moving from the right of the image toward the left at a speed of ca. 4 kts. The white bar represents a seafloor distance of 50 cm. a) Here the net is being used to sample epifauna from a pebble and boulder covered seafloor. b) In many polar regions squid are present in high abundances in the lowest 10 m of the ocean water column. Here a squid is about to be captured with the Niebuhr net. c) Two examples of the icefish *Neopagetopsis ionah*. These fish were quite mobile and attempted to evade the net by swimming directly away from it, in the direction of net travel. The lower of the two fish was captured immediately after this image was taken.

During the 11 deployments made on the expedition, the Niebuhr net was used to collect fish, squid and seafloor fauna from pebble and rock covered areas of seafloor (Fig. 6**a**), areas of old icefish nests with small corals and small pebbles (Fig. 6**b**) and areas of very soft seafloor (Fig. 6**c**). In all cases the net performed very well.

During the expedition the Niebuhr net was generally flown with the welded heavy bottom chain of the net open aperture approximately 10 to 20 cm above the seafloor. When a seafloor target of interest appeared ahead of the nest, the operator would lower the net the few cm to put the welded heavy chain into contact with the seafloor. This would drag the semicircle of welded metal backward and slide it across the seafloor, or into icefish nests if present. Any target fish or fauna unable to avoid capture would then be scooped into the net, past the non-return ring and remain within the net for the rest of the duration of the dive. By actively raising and lowering the net we were able to collect particular fauna and limit the collection of unrequired specimens for our research aims. Additionally, if a large boulder was encountered the Niebuhr net could be winched above the obstacle directly – a facility not offered by traditional seafloor sampling methodologies.

**Fig. 7:**
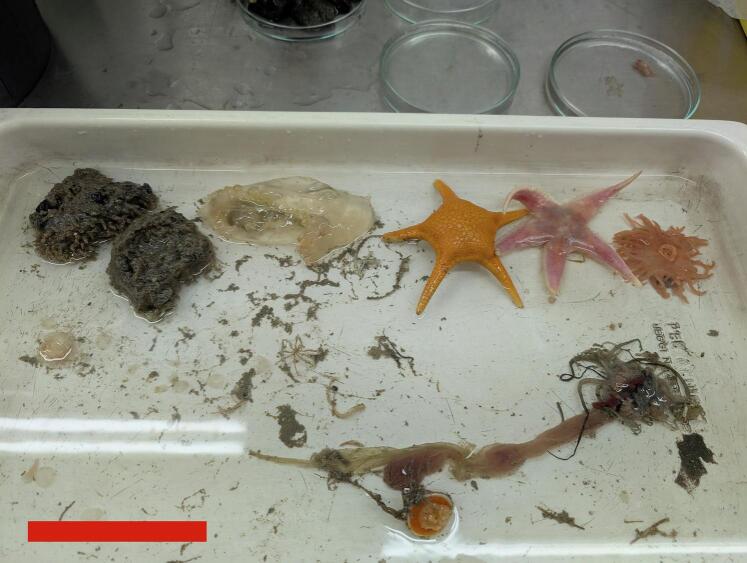
Selection of fauna collected during the deployment shown in Fig. 6a. The red bar represents a distance of 10 cm. Numerous species of anemones, starfish, ophiuroids, molluscs, sponges, worms of various types, holothurians, pychnogonids and krill were all brought to seafloor intact and remained alive in aquaria for the remaining six week duration of the PS146 expedition. (For interpretation of the references to colour in this figure legend, the reader is referred to the web version of this article.)

The dual layer of net appeared to allow collection of many fauna in a good condition, with small, delicate fauna such as sea spiders falling from the wider inner net into the outer net and being brought to the surface with the great majority very active and showing no signs of immediate or future distress. Soft fauna, such as sea cucumbers and some annelid worms were also retrieved in a good condition, as were anemones, starfish, echinoids and urchins, as well as pelagic fauna, such as krill and decapods (Fig. 7), all fauna which can sometimes be damaged by scientific bottom trawling, Agassiz trawling or epibenthic sled trawling.

### 7.3 Conclusions

After extensive use during the challenging PS146 *RV Polarstern* expedition to the ice-covered Weddell Sea, Antarctica, the Niebuhr net has been shown to be a versatile direct seafloor and near seafloor sampling tool for collection of physical fauna samples. The functionality to separate large fauna from smaller fauna on point of collection results in minimal physical damage to the collected fauna on retrieval. The coupling of the Niebuhr net with a towed camera platform allows for the direct sampling of target fauna, a key aim of many research projects where a generalized collection of present fauna is not required. The small aperture of the Niebuhr net, coupled with the live view of the net supplied by the towed camera platform allows the system to sample amongst boulders and challenging seafloor topography that would be unsuitable for sampling by the majority of seafloor sampling devices, such as Agassiz trawl or epibenthic trawl systems. The facility or record collection digitally via the camera sled cameras additionally supports use in vulnerable environments, such as the Weddell Sea or coral reef environments where seafloor impacts are to be avoided.

The low cost, low complexity and ease of building of the Niebuhr net render it an attractive option for physical sampling of the ocean seafloor for a range of researchers. The simplicity of the design allows easy modification for use with different towed camera sleds and to be tailored for collection of different size ranges of target fauna. The articulated attachment arms when coupled with bungee ropes allows a rigidity sufficient to keep the net aperture open whilst mobile enough to allow the net to bounce over obstacles if they cannot be avoided. All of these points highlight a device with numerous research applications and potential interested users.

## Ethics statements

Although the Niebuhr net can be used to collect fauna from aquatic environments, none are harmed in the construction of the net. For the deployment described in section 7 all appropriate permissions for work in Antarctica were collected prior to conducting the work.

## Declaration of generative AI and AI-assisted technologies in the writing process

AI was not used at any stage in the design of the Niebuhr net or for any part in writing this manuscript. The lead author is against using AI in any aspect of scientific paper writing.

## CRediT authorship contribution statement

**Autun Purser:** Writing – original draft, Visualization, Validation, Resources, Project administration, Methodology, Investigation, Conceptualization. **Axel Nordhausen:** Methodology, Conceptualization. **Ulrich Hoge:** Methodology, Conceptualization. **Tim Niebuhr:** Validation, Methodology, Investigation, Conceptualization. **Loki Williams:** Methodology, Conceptualization. **Lisa Chakrabarti:** Investigation, Conceptualization. **Chiara Papetti:** Investigation, Conceptualization. **Malte Pallentin:** Methodology. **Frank Wenzhoefer:** Resources, Conceptualization.

## Funding

The PS146 expedition was funded by AWI Grant No AWI_PS146_04. Chiara Papetti received additional funding from the EU Biodiversa+ WOBEC project. Lisa Chakrabarti was funded by the University of Nottingham. Malte Pallentin was funded by the DLR TRIPLE project.

## Declaration of competing interest

The authors declare that they have no known competing financial interests or personal relationships that could have appeared to influence the work reported in this paper.
